# Implementation of a Budo group therapy for psychiatric in- and outpatients: a feasibility study

**DOI:** 10.3389/fpsyt.2024.1338484

**Published:** 2024-02-02

**Authors:** Jasprit Singh, Karl Jawhari, Mariela Jaffé, Lukas Imfeld, Franziska Rabenschlag, Julian Moeller, André Nienaber, Undine E. Lang, Christian G. Huber

**Affiliations:** ^1^ Universitäre Psychiatrische Kliniken (UPK) Basel, Klinik für Erwachsene, University of Basel, Basel, Switzerland; ^2^ Faculty of Psychology, Division of Clinical Psychology and Epidemiology, University of Basel, Basel, Switzerland; ^3^ Department for Public Mental Health, Zentralinstitut für Seelische Gesundheit, Mannheim, Germany

**Keywords:** mental health, physical exercise, martial arts, group therapy, resilience

## Abstract

**Introduction:**

Physical exercise has been shown to have numerous health benefits on co-morbid somatic conditions in psychiatry and can also enhance mental health. Thus, it is not difficult to recommend physical training programs as part of an integrated and holistic treatment approach for mental health disorders. However, getting patients to participate and keeping them engaged is a major challenge. Programs based on martial arts training could be interventions improving physical and mental health with higher attachment rates. The structured discipline, holistic approach integrating physical and mental elements, and empowering activities, may explain higher participant attachment rates.

**Methods:**

Thus, the main objective of this feasibility study is to describe a newly established group therapy program incorporating interventions from martial arts training with its physical and philosophical parts including mindfulness and breath work.

**Results:**

During the 14-month study period from April 2021 to May 2022, a Budo group therapy was used by 215 individual persons with a total of 725 group therapy participations. Retention in the program was good across all settings and very good for persons who participated as outpatients. The mean age of the participants was 33.5 years with a range from 14 to 69 years of age, and about 41% of the participants were female. The therapy program was able to address patients over the whole spectrum of psychiatric diagnoses. Satisfaction and motivation were uniformly self-reported as very good. Patients self-reported improved mental and physical health after participating in a Budo session compared to pre-session.

**Discussion:**

Budo group therapy thus can be seen as a feasible, well-accepted and promising new transdiagnostic treatment approach, combining physical activation with resilience enhancement. With minimal contraindications, a broad spectrum of individuals seeking mental health support can engage in this group therapy.

## Introduction

Individuals with mental health problems often also have somatic illnesses with a lifetime prevalence of up to 46.6% for any comorbidity (1). Comorbidities can arise as somatic health issues increase in the general population. In particular, cardiovascular disease and ischemic events have been increasing in the general population over the last few decades (2). The same is observable for metabolic syndrome with its defining factors obesity, elevated blood-glucose, insulin resistance, hyperlipidaemia and hypertension (3). Both metabolic syndrome and cardiovascular disease are also leading causes for the premature all-cause mortality in psychiatric patients. These conditions can also arise because of known behavioural risk factors such as smoking, alcohol consumption, poor nutritional diet, poor sleep hygiene and physical inactivity. The risk factors are often observed among psychiatric patients accompanying or independent of the underlying psychiatric conditions (4). In addition, adverse effects of psychopharmacological medication can also contribute to the development or worsening of these comorbidities (5, 6). Vice versa, there are also studies showing that impaired physical health may lead to psychiatric conditions (7).

In this context, physical exercise has been shown to have numerous health benefits. Positive effects can be seen in, e.g., enhancing cardiovascular and cardiorespiratory fitness, preventing or improving metabolic disorders such as insulin resistance, strengthening the immune system and increasing life expectancy (8, 9). According to guidelines for patients with chronic conditions such as cardiovascular disease and metabolic syndrome, the recommended approach for prevention and treatment is increasing regular physical exercise to at least 150-300 minutes of moderate-intensity or 75-150 minutes of vigorous-intensity aerobic physical exercise per week (10, 11). In patients with an elevation in cardiovascular risk, physical exercise has been shown to be advantageous compared to management of cardiovascular disorders and risk factors by medication, leading to an improvement in all-cause-mortality and fewer side effects (12).

However, regular physical activity can also enhance mental health, even having potential benefits equal to psychotherapy (13). Advantageous behavioural changes caused by physical activity and their mechanisms of action are being studied extensively. After single bouts of exercise a decrease in negative and increase in positive mood has been reported lasting up to 24 hours (14). Differing from many health improvements where effects are not observable instantly, e.g. weight loss or cardiovascular fitness, improvements in sleep parameters can be observed the following night directly after the bout of exercise. Furthermore, attending to regular exercise can lead to significant subjective and objective benefits comparable to other interventions used to treat insomnia (15). Emotion regulation is the ability of an individual to influence the occurrence, experience and expression of emotions and therefore includes the awareness of ones feelings. It is a main target of cognitive-behavioural therapy and has been shown to improve through physical activity (16). Delayed gratification by allowing short-term discomfort for long-term benefits is crucial for impulse control and it is imitated and trained by regular physical activity. Many important self-regulatory processes are combined when attending to physical activity such as setting goals, activity planning and self-monitoring (17). In a meta-analytic review on physical exercise and its effects on cognitive performance, the positive influence of different levels of exercise intensities was described. Lower intensities were linked with improvements in cognition immediately following the bout of exercise, whereas after more strenuous bouts beneficial effects were observable for longer durations (18). Another behavioural amelioration that has been reported due to exercise is in cognitive flexibility (17), i.e., the capacity of a person to adjust their thoughts and actions according to varying circumstances (19). These benefits can be further strengthened by incorporating, e.g., mindfulness-based interventions and meditation into the physical training program (16, 20, 21). Finally, due to the interdependence of physical and mental health and the high degree of comorbidities, benefits in one health area are also suitable to improve health in the other (17, 22).

In light of the vast positive impact of physical exercise on many risk factors and overall health, it is evidence-based to recommend physical training programs as part of an integrated and holistic treatment approach for mental health disorders. However, getting people to participate and keeping them engaged in regular physical activity is a major challenge, making this a main obstacle for the success of training programs (23).

Training programs based on martial arts could be interventions improving physical and mental health with higher attachment rates. In a systematic review on the effect of combat sports in middle-aged and older people, the authors observed an adherence rate of greater than 80% in ten different studies. Furthermore, they found an adherence rate between 71 and 96% in 5 different studies in which one was investigating the benefits of boxing, one of judo and three of karate programs (24). Martial arts training seems suitable as an intervention in persons with mental health problems. It has been described to not only bring physical benefits such as improvements in strength, mobility, flexibility and aerobic endurance (25), some forms of training also incorporate mindfulness (26). In a cross-sectional study Miyata et al. showed that practitioners of traditional Japanese martial arts have a proclivity to mindfulness and to better mental health outcomes (27). Other studies show an association of martial arts training and improvement in mindfulness, self-control and wellbeing, and in depressive and anxiety disorders (28). In particular, Budo – known as Japanese martial arts – is getting more attention in the medical field due to its effects on physical and mental health. There are many different subcategories in Budo and the term is not closely defined. It is also used to describe a culture of spirituality and moral values (29). Some subcategories of Budo use weapons and some are solely depending on the capabilities of the human body. However, every Budo -style has the same underlying principles in which it differs from other western recreational sports; Japanese martial arts are moulded by tradition and religion (30). By focussing on calming the mind on the basis of *Fudoshin* (unmoving mind, immovable heart) which is seen as equanimity in Japanese tradition, Budo also encompasses meditational components of Buddhism and Shintoist and Taoist concepts (31, 32). Although having many similarities with other sports, Budo is seen as a method to develop human character and emphasizes on educational aspects (33).

However, and despite the evidence for their suitability for psychiatric patients, literature on combined physical and mental health interventions based on martial arts training in psychiatry is sparse and further research is needed. Following this line of thought, we established a Budo group therapy targeting physical and mental health improvement. By integrating physical exercise, meditational introspection, mindfulness and its moral teachings, we wanted to improve physical health, alleviate psychiatric symptoms and improve the course of the psychiatric conditions of our in- and outpatient participants.

## Aim of the study

Thus, the main objective of the current paper is to describe a newly established Budo group therapy program incorporating interventions from martial arts training with its physical and philosophical parts including mindfulness and work of breathing. We want to assess patients’ usage behaviour and satisfaction with the group therapy, examine the subjective impact on the mental and physical wellbeing of the participants, and gain insight into patient preferences regarding form and frequency of service delivery.

## Subjects and methods

### General framework

The Department of Psychiatry, University of Basel (Universitäre Psychiatrische Kliniken, UPK) in Switzerland is the only psychiatric university hospital of northwestern Switzerland. It provides in- and outpatient services for the city of Basel and the surrounding area with a population of over 200,000 persons. There are around 300 beds available to patients at the UPK Basel in four specialized clinics: the adult psychiatric clinic (UPKE), the private clinic (UPKP), the clinic for children and adolescents (UPKKJ), and the forensic psychiatric clinic (UPKF) for adolescents and adults. The adult psychiatric clinic is organized following a track concept and includes a department for outpatient care, diagnostics and crisis intervention (ZDK), a department for psychotic disorders (ZPE), a department for psychosomatic medicine and psychotherapy (ZPP), a department for affective disorders (ZASS), a department for substance use disorders (ZAE), and a gerontopsychiatric department (ZAP). Apart from psychopharmacological, biological, and psychotherapeutic treatment, the therapy of in- and outpatients consists of various other treatment options, such as occupational therapy, art and gestalt therapy, music therapy, aromatherapy, physiotherapy, and nature-oriented therapy with animals or with gardening activities. In order to improve treatment options, different group therapies are established and evaluated routinely. In April 2021, Budo was implemented in the general therapeutic setting at the UPK as a new therapy group.

### Budo group therapy

The two key elements of Budo – building physical strength and developing spiritual and mental well-being – represent the fundamental basis of each therapy session. Every session has a therapeutic theme that is integrated into the individual training parts. Up to 10 participants were allowed per therapy session. Each training is run by two trainers and is structured in four parts beginning with a greeting, and then a warm-up, followed by theme-oriented exercises. The sessions end with a short debriefing.

*Greeting:* The participants stand side by side and the trainers position themselves in front of them. The trainers signal the start of the session with the first exercise. Everyone closes their eyes, and concentrates on the here and now and on their own body. The participants should allow themselves to feel their current emotions and observe how they feel in their body. This introspection lasts for approximately one minute and is followed by a greeting, brief description of the program and the rules of the group session. These rules can be derived from the main principals of “Syugyo” (life training/self-cultivation) (33) as well as the Japanese etiquette “reigi” (34), and are especially important in the psychiatric setting. The Japanese philosopher Yuasa Yasuo elaborates on this concept of self-cultivation in his book “*The Body, Self-Cultivation, and Ki Energy*” (35). The key rules are: every interaction needs to be polite and respectful; everybody has to pay attention to tidiness and hygiene; everyone has to pay attention to their emotional and physical wellbeing and if they worsen the trainer must be informed; participants are asked to act in a calm attentive manner, heed the advice of the trainer, and never act vengefully. These listed rules should also guide the participants in their day-to-day life even after the therapy session.

*Warm-up*: The warm-up consists of various short mobility and aerobic exercises followed by 15 to 20 minutes stretching. First, the participants have to jog or jump rope for several minutes to stimulate their cardiovascular system. Afterwards they practice their mobility by moving different body parts such as hips, arms, spine, head in a coordinated circular motion thus loosening the big joints and the surrounding muscle groups. Depending on the program, the participants are instructed to imitate the gait of different animals. The trainer demonstrates how this should look and the participants try to re-enact the motion. Shortly before the stretching, the basic kickboxing posture and stances as well as basic kicks are focused on. In a playful manner, participants learn how to move and position themselves. Kickboxing was chosen for its effectiveness in providing a comprehensive physical workout, including cardiovascular exercise and strength training. The dynamic and engaging nature of kickboxing, along with its potential to empower individuals and boost confidence, aligns with many of the goals of the Budo group therapy program. The adaptability of kickboxing to various fitness levels and its popularity as a contemporary fitness activity was thought to maybe have a broad appeal for a wide audience. The warm-up ends with 15 to 20 minutes of static stretching. The body parts that are mainly focused on are the legs and hip region targeting the flexors and extensors as well as the neck, upper back and shoulder musculature. Each stretch is held for at least 30 seconds and is accompanied with breathing instructions.

*Theme-oriented exercises:* The themes of every therapy session can differ. The trainer decides prior to planning a training on what they want to focus. Concentration and attentiveness, tension and relaxation, perceiving and regulating emotions or self-efficacy and self-confidence are themes implemented on a regular basis in each course session. An example for this part on concentration and attentiveness might look like this:

The participants are briefly given a short theoretical background for the exercises. For example: “In some mental illnesses, but also in situations that we are all familiar with from everyday life, concentration and attention can be impaired.” The group is then asked whether anyone has already experienced problems with concentration and attention in their day-to-day life. If so, what was the reason? Afterwards, the task is explained step by step using a flipchart. After the instruction, there is time for questions.

Participants are told that an exercise is being carried out in groups of two and that it should have a positive effect on attention and concentration. Afterwards, the group can share how their experience of the exercise was. One person takes on the role of the coach and takes a large punching pad. The second person starts as the pupil and walks loosely in a circle around the coach. The coach can give three different instructions.

When the coach says “1”, the pupil runs to the punching pad as quickly as possible and performs three punches. When the coach says “2”, the pupil runs as fast as possible to the punching pad and performs a front kick. When the coach says “3”, the pupil runs as fast as possible to the punching pad and jumps as high as possible in front of it. The instructors demonstrate these movements.

The coach has the task of calling out the numbers in any order and at different intervals to check whether the pupils are focusing, and they have to check if they are performing the task correctly. The task takes three minutes, after which the roles are swapped.

If someone notices that they are very tired or not feeling well, they can take a break at any time. They can also ask for help at any time if difficulties arise. They are told that it is important to reduce distractions. The participants are informed about the time frame of the exercise and that there is a break zone. If there are no more questions, the participants are asked to briefly think of the numbers 1, 2 and 3 and to visualize which movement belongs to which number. After the introduction, the exercise begins.

*Debriefing:* The end of the session is structured similarly to the beginning. The participants line up in front of the trainers and everyone closes their eyes at the trainers’ signal. The room gets quiet and everyone visualizes what they learned and practiced in the session. Participants are asked to think of the things they appreciated during the training and their achievements, if there were new techniques or inputs they especially liked or if they maybe feel more energized. They get encouraged that even if they experienced hardships during the day, they finished a strenuous training. Once again, they get the time for introspection, to focus on how they feel and to monitor if there are changes in their self-perception. The debriefing lasts for approximately five minutes. Afterwards, the trainers thank the patients for their participation and the session ends.

### Study population

All in- and outpatients of the adult psychiatric clinic (UPKE/UPKP), the forensic psychiatric clinic (UPKF), and the child and youth psychiatric clinic (UPKKJ) were eligible for participation in the group therapy. The responsible physician had to approve an individual patient’s suitability to be enrolled in the Budo group. The study population was not restricted to patients with specific diagnoses. Exclusion criteria were recent cardiovascular or orthopaedic interventions, and general medical illness, e.g., flu-like symptoms. Patients with any sort of acute somatic pain, except for muscle tension, were also excluded.

### Participant survey

On the basis of our clinic-wide quality management concept following the PDCA (Plan – Do – Check – Act) cycle in implementing, monitoring, and improving new therapies, the participants were able to fill in a survey after each session. They were able to answer questions about their wellbeing and whether or not they see an additional benefit by attending the Budo group. Details on the questionnaire are available in the online-only supplement to this article. Participants had the option to provide identifying data, enabling the tracking of participating multiple times in the survey and the pseudo-anonymized extraction of routine clinical data, or to participate anonymously. The survey was conducted from April 2021 until May 2022 using EFS Survey/Unipark (Tivian XI GmbH, Cologne, Germany).

### Documentation and management of clinical data

Clinical and treatment data were continuously documented using the Medfolio software (current version: 2.2.0.2539, release sr27qs1.0sp_kged32_560; NEXUS AG, Villingen-Schwenningen, Germany) and extracted using HCe^®^ Analytics software (Business Intelligence Connector 3 (BIC 3) for patient controlling; TIP Management AG, Dübendorf, Switzerland). Data on age, gender, diagnoses according to the International Classification of Diseases, 10th revision (ICD-10 (WHO, 1992)), and legal status were documented by the psychiatrists responsible for the respective patient.

As data was documented during routine treatment and anonymized during data extraction, and as the survey was performed within the clinic’s routine quality management assessments, data collection and analyses were exempt from ethics committee approval. Publishing the results was not pre-planned, but decided on afterwards to facilitate the transfer of our experiences into clinical practice. The local ethics committee (EKNZ; Ethik-Kommission Nordwest- und Zentralschweiz) has provided a written confirmation that no ethics committee vote was needed for this publication (Req-2023-00460).

### Statistical analysis

Participation in the group therapy, clinical and sociodemographic characteristics, and variables from the survey are given in total numbers and percentages for nominal scaled variables as well as mean and standard deviation (SD) for ordinal and interval scaled variables. This being a feasibility study, and because of the relatively modest sample size of the survey, supportive explorative statistical analyses are reported for descriptive purposes only. Statistical analyses were conducted using IBM SPSS Statistics for Windows, Version 27.0 (Released 2020. IBM Corp. Armonk, NY) as well as R-Studio, Version 2023.03.1 (R Foundation for Statistical Computing, Vienna, Austria).

## Results

During the 14-month study period from April 2021 to May 2022, the Budo group therapy was used by 215 individual persons with a total of 725 group therapy participations. Patients took part in different treatment settings (i.e., inpatient or outpatient treatment) and each admission to a treatment setting is documented as a separate case, resulting in 236 treatment cases. Of these, 214 cases took part during inpatient treatment and had 2.6 ± 2.2 (mean ± SD) treatment sessions (range: 1 – 13 sessions); 16 during outpatient treatment with 7.2 ± 14.2 treatment sessions (range: 1 – 49); and six took part both during in- and outpatient treatment with 9.4 ± 3.0 treatment sessions (range: 6 – 13).

Clinical and sociodemographic data of group therapy and survey participants are presented in **Table 1**. With 87%, most group therapy participants were patients from the adult psychiatric clinic (UPKE). About one in ten participants was a patient from the private clinic (UPKP) attended, and single participants from the clinic for children and adolescents (UPKKJ) as well as from the forensic psychiatric clinic (UPKF). Within the UPKE, all diagnostic departments contributed patients with the exception of the department for gerontopsychiatry (ZAP). Mean age of the participants at entry was 33.5 years with a range from 14 to 69 years of age, and about 41% of the participants were female. About 4% of the participants had been involuntarily admitted to general or forensic psychiatric inpatient treatment and the same percentage of participants had been subjected to seclusion or involuntary treatment. Most participants had a main diagnosis of affective disorders (37%) or substance use disorders (23%).

**Table 1:** Clinical and sociodemographic data of group therapy and survey participants.

	Group therapy (*n* = 215)	Survey (*n* = 46)
Psychiatric clinic or department^a,b^		
Adult psychiatric clinic (UPKE)	187 (87%)	40 (87%)
UPKE Department for outpatient treatment and crisis intervention (ZDK)	23 (10.7%)	5 (10.9%)
UPKE Department for psychotic disorders (ZPE)	26 (12.1%)	3 (6.5%)
UPKE Department for substance use disorders (ZAE)	44 (20.5%)	7 (15.2%)
UPKE Department for psychosomatic medicine and psychotherapy (ZPP)	40 (18.6%)	12 (26.1%)
UPKE Department for affective disorders (ZASS)	54 (25.1%)	13 (28.3%)
UPKE Department for gerontopsychiatry (ZAP)	0 (0%)	0 (0%)
Private clinic (UPKP)	22 (10.2%)	6 (13%)
Clinic for children and adolescents (UPKKJ)	1 (0.5%)	0 (0%)
Forensic psychiatric clinic (UPKF)	5 (2.3%)	0 (0%)
**Duration of inpatient treatment (mean ± SD)^c^**	81.1 ± 132.3	73.7 ± 62.1
Involuntary measure		
Involuntary entry	8 (3.7%)	0 (0%)
Seclusion and/or involuntary treatment	8 (3.7%)	0 (0%)
Sociodemographic data^a,d^		
Age at entry (mean ± SD)	33.5 ± 11.9	31.9 ± 10.4
Female gender (%)	89 (41.4%)	20 (43.5%)
Main diagnosis (ICD-10)^e,f^		
Organic, including symptomatic, mental disorders(F0)	1 (0.5%)	1 (2.2%)
Mental and behavioural disorders due to psychoactive substances (F1)	49 (22.8%)	10 (21.7%)
Schizophrenia, schizotypal and delusional disorders (F2)	17 (8%)	0 (0%)
Mood, affective disorders (F3)	80 (37.2%)	19 (41.3%)
Neurotic, stress-related and somatoform disorders (F4)	30 (14%)	7 (15.2%)
Behavioural syndromes with physiological disturbances and physical factors(F5)	3 (1.4%)	0 (0%)
Disorders of adult personality and behaviour (F6)	28 (13%)	6 (13%)
Mental retardation (F7)	0 (0%)	0 (0%)
Disorders of psychological development (F8)	1 (0.5%)	0 (0%)
Behavioural and emotional disorders, onset in childhood or adolescence (F9)	4 (1.9%)	1 (2.2%)

^a^no missing data; ^b^in general, about 88% of all cases are treated in the UPKE, 6% in the UPKP, 4% in the UPKKJ, and 2% in the UPKF, missing data; ^c^missing data for 11 group therapy participants (for involuntary entry variable only) and for one survey participants; ^d^with about 48% of all patient cases being of female gender; ^e^missing data for two group therapy participants and for two survey participants; ^f^distribution of main diagnoses for all patients in the hospital in 2022 was: F0: 1.5%, F1: 24.5%, F2: 17.6%, F3: 25.7%, F4: 15.4%, F5: 0.5%, F6: 11.5%, F7: 0.5%, F8: 0.3%, F9: 1.4%, others: 1.2%.

While survey participants were – in general – comparable to group therapy participants, no patients from the UPKKJ and the UPKF took part in the survey. Also, persons with involuntary entry, exposed to seclusion or involuntary treatment, or diagnosed with schizophrenia spectrum disorders were missing from the survey.

Exploratory analyses showed no evidence for significant statistical correlations between the frequency of group therapy attendance and gender, age, and duration of inpatient treatment (all *r* < |.12|; *p* >.110).

74 participants filled in the survey in the study period of April 2021 to May 2022. Results are presented in **Table 2**. Using a 5-point Likert scale with a rating of 1 indicating lowest rating or agreement and 5 indicating highest rating or agreement, participants showed high values for satisfaction (4.9 ± 0.3, range 4 – 5) and motivation (4.8 ± 0.4, range 4 – 5).

**Table 2:** Survey on self-reported satisfaction and motivation, intervention effects, and perspectives on future use.

	Participants		Male^a^		Female^a^		*p-value*
	*n*	*M ± SD*	*n*	*M ± SD*	*n*	*M ± SD*	
Satisfaction and motivation							
How satisfied were you with the Budo group therapy?	73	4.9 *±* 0.3	36	4.9 *±* 0.2	27	5.0 *±* 0.2	p = .738
How motivated were you during the Budo therapy session?	73	4.8 *±* 0.4	36	4.8 *±* 0.4	27	4.9 *±* 0.4	p = .638
Intervention effects							
How do you feel physically?	30	4.3 *±* 0.8	14	4.6 *±* 0.6	11	4.0 *±* 0.8	p = .056
How do you feel psychologically?	30	4.1 *±* 0.9	14	4.6 *±* 0.5	11	3.6 *±* 0.7	p <.001
Perspectives on future use							
Do you think that the Budo group therapy is beneficial in the therapeutic setting?	72	4.8 *±* 0.4	35	4.9 *±* 0.4	27	4.8 *±* 0.5	p = .354
Would you like to use the Budo group therapy multiple times per week?	72	4.6 *±* 0.8	36	4.8 *±* 0.6	26	4.4 *±* 0.9	p = .081
Do you think that the Budo group therapy would be beneficial even after discharge from inpatient treatment?	67	4.6 *±* 0.7	34	4.6 *±* 0.7	24	4.6 *±* 0.6	p = .838

All ratings were made on a 5-point Likert scale, with 1 indicating lowest rating or approval and 5 indicating highest rating or approval; ^a^self-identified gender.

Patients self-reported that they felt markedly better physically (4.3 ± 0.9, range 1 – 5) and psychologically (4.1 ± 0.8, range 2 – 5) after group therapy participation. While most participants reported an improvement, there was a small number of participants who felt worse physically (3.3% rated “physically, I feel a lot worse”) or psychologically (6.7% rated “psychologically, I feel worse”). Comparing female and male participants, exploratory analyses indicated that male participants might report better subjective improvement physically (*t*(23) = 2.01, *p* = .056) and psychologically (*t*(23) = 4.30, *p* = <.001) compared to female participants. Furthermore, Pearson analyses indicated a positive correlation of psychological (*r* = .544, *p* = .036), but not physical improvement (*r* = -.172, *p* = .540) with participant age. Feeling psychologically better was highly correlated with feeling physically better (*r* = .609, *p* <.001).

Concerning future use of the group therapy, survey participants indicated that they felt Budo was an important addition to the therapeutic programme (4.8 ± 0.4, range 3 – 5), that they were interested in partaking multiple times a week (4.6 ± 0.8, range 2 – 5), and that they would like to continue participating after discharge from an inpatient setting (4.6 ± 0.7, range 2 – 5).

## Discussion

In this feasibility study, we investigated the practical implementation of a Budo group therapy program in a subset of psychiatric in- and outpatients. In addition to information on group therapy participation and retention, a modest retrospective observational analysis was conducted to provide further information on satisfaction and motivation, subjective self-reported intervention effects, and perspectives on future use. Strengths of this study are the novelty of the treatment approach, presenting naturalistic usage data from a large psychiatric university hospital covering the whole spectrum of patients, and the inclusion of subjective participant feedback.

Usage data show that the Budo group therapy could be successfully introduced and was accepted very well. A considerable number of patients participated in the sessions. Although most patients participated from an inpatient treatment setting, some patients started to participate in an outpatient treatment setting or continued to participate after discharge from inpatient treatment. Retention in the program was good overall with a mean of over three participations per person, and very good in persons who participated as outpatients (with a mean of over seven participations) or as in- and outpatients (with a mean of over nine participations). This is further supported by the responses in the patient survey concerning perspectives of future use, indicating favourable ratings regarding the importance of the treatment approach, the interest in continuing to participate after discharge, and the interest in partaking multiple times a week. These results are compatible with findings of a high adherence to martial arts based exercise therapy (24). Thus, Budo group therapy could be a promising candidate for a combined therapeutic intervention aimed at mental and physical health with high adherence after hospitalisation and could help integrate physical exercise in patients’ lives in the long run.

The group therapy program was able to address patients from all parts of the hospital with the exception of the department of gerontopsychiatry. Considering the overall case distribution in the hospital (as provided in detail in the legend for **Table 1**), there seems to be an overrepresentation of participants from the private clinic and an underrepresentation of patients from the clinic for children and adolescents. As apt for a transdiagnostic treatment approach, participants had main diagnoses covering the whole spectrum of psychiatric disorders. Considering the overall case distribution in the hospital (as provided in detail in the legend for **Table 1**), there seems to be a descriptive underrepresentation of patients with schizophrenia spectrum disorders and a descriptive overrepresentation of patients with affective disorders. No patients with mental retardation took part, which is unsurprising as they are rarely admitted to this institution. Both male and female patients used the Budo group therapy program, with numbers suggesting only a slight descriptive underrepresentation of female participants. As – in theory – a group therapy including elements from martial arts training might attract a predominantly male audience, this is, however, quite encouraging. While mean age suggests that younger persons were descriptively overrepresented, participants in general covered a broad age range from 14 to 69, suggesting that the Budo group therapy might also be suitable for older adult persons. Nevertheless, missing participation from patients from the department of gerontopsychiatry indicates that appropriateness of the therapy program for persons with an age of 70 and upwards, with a diagnosis of dementia and with severe somatic co-morbidities should be further assessed.

Judging from the data of persons allowing identification of their clinical information, survey participation seems to be in general representative of the participant group. However, no participants from the forensic clinic, the clinic for children or adolescents, diagnosed with a schizophrenia spectrum disorder or having been involuntarily admitted or subjected to seclusion or coercive medication could be identified. It remains unclear if these patient groups did not participate in the survey or if they are part of the about one third of the participants that did not allow linking their clinical data.

Satisfaction and motivation were uniformly rated very highly by all patients participating in the survey, with ratings only indicating either high or very high satisfaction and motivation. This has to be seen in the context that the group therapy was not mandatory and might have primarily attracted persons that were prone to exercise and therefore to positive ratings. However, the large number of participants and the missing of any negative or neutral ratings suggest that the positive ratings may not only be due to participant self-selection. An increase in life satisfaction in people engaging in other martial arts has been observed as well. In a systematic review on Brazilian jiu-jitsu and its effects as social and psychological therapy the author elaborated on increased satisfaction and pro-social behaviour and reduced symptoms of various psychiatric conditions in participants of Brazilian jiu-jitsu. Furthermore, he describes a social benefit with a sense of community and belonging (36). The participants of the Budo group therapy may experience similar effects, which could explain these high ratings. Intervention effects were self-reported as generally positive in mental and physical health, with favourable mean ratings and most patients reporting improvement of their health. Only one in 30 participants described feeling worse physically, and about one in 16 participants did feel worse psychologically.

Exploratory analyses suggest that male participants might subjectively experience more physical and psychological improvement from the therapy sessions than female participants, and that higher age might be connected with increased psychological improvements. Our findings on a putative difference could be between female and male participants’ subjective physical and psychological improvements are compatible with the current literature. For example, Tiggemann and Williamson showed that men who engage in high intensity exercise report greater psychological improvements especially in self-esteem compared to women (37). Furthermore, the gender difference is associated with different motives to engage in exercise. Craft et al. illustrated that unrelated to the underlying reason for physical exercise, men showed a higher quality of life whereas in women the motive for exercising was important in the context of quality of life (38). Nonetheless, the number of female participants and their subjective improvements show the positive potential of this group therapy in male and female participants. As Kavoura et al. discussed, there are numerous factors for underrepresentation of female martial artists in psychological research such as historical bias, gender stereotypes and many more (39). Therefor it is crucial not to disregard the results of the female participants. However, given the preliminary nature of the survey and the small sample size, our findings have to be interpreted very cautiously.

The effects of physical exercise and of mindfulness based interventions on stress are well known (13). Budo based therapy combines both and may therefore also help prevent or reduce stress, agitation and aggression, which can lead to involuntary measures in psychiatry (40, 41). In a review on social-psychological outcomes of martial arts practise among youth contrasting findings were discussed concerning the effects of martial arts training on aggression. Some studies described positive outcomes with decreases in hostility and aggression and better self-control, whereas others reported negative effects such as more antisocial behaviour (42). In that review, however a different age group was focused on with no known psychiatric conditions, which makes it difficult to draw conclusions for the participants of the Budo group therapy. While the Budo group therapy was able to successfully address patients with main diagnoses often associated with aggression and coercion in psychiatry (i.e., ICD-10 main diagnoses from chapters F0, F1, F2, F3, and F6), patients from forensic psychiatry settings, and patients subjected to involuntary admission, seclusion, and involuntary medication, the number of these participants was limited, and no outcomes addressing stress levels, agitation and aggression were available. Therefore, the potential of Budo group therapy sessions to aid in dealing with agitation and aggression in psychiatry remains to be seen and should be explored in future research.

## Limitations

There was no pre-established study protocol, therefore resulting in limited depth of information available for analysis. The survey data primarily relied on self-reported responses from individuals who had participated in the program and hence experienced the new treatment, potentially introducing a bias towards more favourable assessments. Nonetheless, the substantial interest and engagement of the patients indicated a widespread interest in this new treatment approach. However, future research should consider the use of randomized allocation and establishment of a control group to address potential biases and provide comparisons that are more robust. Regardless, it is worth noting that the novelty of the treatment option and the results indicating its high potential emphasise the need for further research in this area.

## Conclusion

Budo group therapy is feasible in an inpatient and outpatient psychiatric setting and offers a unique approach in the possibilities of therapeutic interventions in mental health. Its ability to combine physical activation with resilience enhancement provides a powerful instrument for the promotion in mental well-being. Furthermore, it has a transdiagnostic applicability allowing patient participation across a wide range of psychiatric conditions. With minimal contraindications, a broad spectrum of individuals seeking mental health support can engage in the group therapy. Most notably, Budo has a capacity to motivate patients, fostering a sense of empowerment and self-improvement. As research continues to search for innovative interventions in mental health treatment, the integration of martial arts stands as a compelling option, worthy of further investigation and incorporation into therapeutic practices.

## Data availability statement

The original contributions presented in the study are included in the article/**Supplementary Material**. Further inquiries can be directed to the corresponding author.

## Ethics statement

The Ethics Committee Northwest Switzerland EKNZ declared that no ethic committee approval was needed for the current study (Req-2023-00460). The studies were conducted in accordance with the local legislation and institutional requirements. Written informed consent for participation was not required from the participants or the participants’ legal guardians/next of kin in accordance with the national legislation and institutional requirements.

## Author contributions

JS: Writing – original draft. KJ: Conceptualization, Writing – original draft. MJ: Formal analysis, Writing – original draft. LI: Data curation, Writing – review & editing. FR: Project administration, Writing – review & editing. JM: Supervision, Writing – review & editing. AN: Supervision, Writing – review & editing. UL: Supervision, Writing – review & editing. CH: Conceptualization, Supervision, Writing – original draft.

## References

[1] Finlay S Rudd D McDermott B Sarnyai Z. Allostatic load and systemic comorbidities in psychiatric disorders. Psychoneuroendocrinology (2022) 140:105726. doi: 10.1016/j.psyneuen.2022.105726 35339811

[2] Roth GA Mensah GA Johnson CO Addolorato G Ammirati E Baddour LM. Global burden of cardiovascular diseases and risk factors, 1990–2019: update from the GBD 2019 study. J Am Coll Cardiol (2020) 76(25):2982–3021. doi: 10.1016/j.jacc.2020.11.010 33309175 PMC7755038

[3] Eckel RH Grundy SM Zimmet PZ. The metabolic syndrome. Lancet (2005) 365(9468):1415–28. doi: 10.1016/S0140-6736(05)66378-7 15836891

[4] Penninx B Lange SMM. Metabolic syndrome in psychiatric patients: overview, mechanisms, and implications. Dialogues Clin Neurosci (2018) 20(1):63–73. doi: 10.31887/DCNS.2018.20.1/bpenninx 29946213 PMC6016046

[5] Mackin P. Cardiac side effects of psychiatric drugs. Hum Psychopharmacol (2008) 23 Suppl 1:3–14. doi: 10.1002/hup.915 18098218

[6] Schwartz TL Nihalani N Jindal S Virk S Jones N. Psychiatric medication-induced obesity: a review. Obes Rev (2004) 5(2):115–21. doi: 10.1111/j.1467-789X.2004.00139.x 15086865

[7] Barnett K Mercer SW Norbury M Watt G Wyke S Guthrie B. Epidemiology of multimorbidity and implications for health care, research, and medical education: a cross-sectional study. Lancet (2012) 380(9836):37–43. doi: 10.1016/S0140-6736(12)60240-2 22579043

[8] Reimers CD Knapp G Reimers AK. Does physical activity increase life expectancy? A review of the literature. J Aging Res (2012) 2012:243958. doi: 10.1155/2012/243958 22811911 PMC3395188

[9] Ruegsegger GN Booth FW. Health benefits of exercise. Cold Spring Harb Perspect Med (2018) 8(7). doi: 10.1101/cshperspect.a029694 PMC6027933 28507196

[10] Okely AD Kontsevaya A Ng J Abdeta C. 2020 WHO guidelines on physical activity and sedentary behavior. Sports Med Health Sci (2021) 3(2):115–8. doi: 10.1016/j.smhs.2021.05.001 PMC9219310 35782159

[11] Dobrowolski P Prejbisz A Kuryłowicz A Baska A Burchardt P Chlebus K. Metabolic syndrome - a new definition and management guidelines: A joint position paper by the Polish Society of Hypertension, Polish Society for the Treatment of Obesity, Polish Lipid Association, Polish Association for Study of Liver, Polish Society of Family Medicine, Polish Society of Lifestyle Medicine, Division of Prevention and Epidemiology Polish Cardiac Society, “Club 30” Polish Cardiac Society, and Division of Metabolic and Bariatric Surgery Society of Polish Surgeons. Arch Med Sci (2022) 18(5):1133–56. doi: 10.5114/aoms/152921 PMC9479724 36160355

[12] Pozuelo-Carrascosa DP Cavero-Redondo I Rodríguez RF Morena CP Sequí-Domínguez I Martinez-Vizcaino V. Exercise versus fixed-dose combination therapy for cardiovascular risk factors control and atherosclerotic disease prevention: a network meta-analysis protocol. BMJ Open (2020) 10(7):e036734. doi: 10.1136/bmjopen-2019-036734 PMC7348467 32641333

[13] Paluska SA Schwenk TL. Physical activity and mental health: current concepts. Sports Med (2000) 29(3):167–80. doi: 10.2165/00007256-200029030-00003 10739267

[14] Basso JC Suzuki WA. The effects of acute exercise on mood, cognition, neurophysiology, and neurochemical pathways: A review. Brain Plast (2017) 2(2):127–52. doi: 10.3233/BPL-160040 PMC5928534 29765853

[15] Kredlow MA Capozzoli MC Hearon BA Calkins AW Otto MW. The effects of physical activity on sleep: a meta-analytic review. J Behav Med (2015) 38(3):427–49. doi: 10.1007/s10865-015-9617-6 25596964

[16] Zhang Y Fu R Sun L Gong Y Tang D. How does exercise improve implicit emotion regulation ability: preliminary evidence of mind-body exercise intervention combined with aerobic jogging and mindfulness-based yoga. Front Psychol (2019) 10:1888. doi: 10.3389/fpsyg.2019.01888 31507480 PMC6718717

[17] Smith PJ Merwin RM. The role of exercise in management of mental health disorders: an integrative review. Annu Rev Med (2021) 72:45–62. doi: 10.1146/annurev-med-060619-022943 33256493 PMC8020774

[18] Chang YK Labban JD Gapin JI Etnier JL. The effects of acute exercise on cognitive performance: A meta-analysis. Brain Res (2012) 1453:87–101. doi: 10.1016/j.brainres.2012.02.068 22480735

[19] Uddin LQ. Cognitive and behavioural flexibility: neural mechanisms and clinical considerations. Nat Rev Neurosci (2021) 22(3):167–79. doi: 10.1038/s41583-021-00428-w PMC7856857 33536614

[20] Moore A Malinowski P. Meditation, mindfulness and cognitive flexibility. Consciousness Cogn (2009) 18(1):176–86. doi: 10.1016/j.concog.2008.12.008 19181542

[21] Herwig U Opialla S Cattapan K Wetter TC Jäncke L Brühl AB. Emotion introspection and regulation in depression. Psychiatry Res: Neuroimaging (2018) 277:7–13. doi: 10.1016/j.pscychresns.2018.04.008 29778804

[22] Golbidi S Mesdaghinia A Laher I. Exercise in the metabolic syndrome. Oxid Med Cell Longev (2012) 2012:349710. doi: 10.1155/2012/349710 22829955 PMC3399489

[23] Collado-Mateo D Lavín-Pérez AM Peñacoba C Coso Del J Leyton-Román M Luque-Casado A. Key factors associated with adherence to physical exercise in patients with chronic diseases and older adults: an umbrella review. Int J Environ Res Public Health (2021) 18(4). doi: 10.3390/ijerph18042023 PMC7922504 33669679

[24] Valdés-Badilla P Herrera-Valenzuela T Guzmán-Muñoz E Delgado-Floody P Núñez-Espinosa C Monsalves-Álvarez M. Effects of olympic combat sports on health-related quality of life in middle-aged and older people: A systematic review. Front Psychol (2021) 12:797537. doi: 10.3389/fpsyg.2021.797537 35069389 PMC8769282

[25] Miller I Climstein M Vecchio LD. Functional benefits of hard martial arts for older adults: A scoping review. Int J Exerc Sci (2022) 15(3):1430–43. 10.70252/WZQA6910 PMC9797003 36618333

[26] Naves-Bittencourt W Mendonça-de-Sousa A Stults-Kolehmainen M Fontes E Córdova C Demarzo M. Martial arts: mindful exercise to combat stress. Eur J Hum Mov (2015) 34:34–51.

[27] Miyata H Kobayashi D Sonoda A Motoike H Akatsuka S. Mindfulness and psychological health in practitioners of Japanese martial arts: a cross-sectional study. BMC Sports Sci Med Rehabil (2020) 12(1):75. doi: 10.1186/s13102-020-00225-5 33372628 PMC7720472

[28] Moore B Dudley D Woodcock S. The effect of martial arts training on mental health outcomes: A systematic review and meta-analysis. J Bodyw Mov Ther (2020) 24(4):402–12. doi: 10.1016/j.jbmt.2020.06.017 33218541

[29] Sasaki T. <Budo (the martial arts) as Japanese culture. The.pdf>. Archives of Budo, Taketo Sasaki (2008). (Fukushima: Faculty of Human development and Culture Department of Physical Education, Fukushima University), 4.

[30] Nakiri F. Concept of budo and the history and activities of the Japanese Academy of Budo. Ido Mov Culture J Martial Arts Anthropol (2015) 15(1):11–25.

[31] Fujiwara H Ueno T Yoshimura S Kobayashi K Miyagi T Oishi N. Martial arts “Kendo” and the motivation network during attention processing: an fMRI study. Front Hum Neurosci (2019) 13. doi: 10.3389/fnhum.2019.00170 PMC6539200 31191277

[32] Cynarski WJ. Asian Martial Arts and its connections with metaphysics or spiritual element. Kampf- und Bewegungskünste (2023) ( Albany : State University of New York Press, USA), 1–8. doi: 10.3389/fnhum.2019.00170

[33] Sasaki T. The meaning and role of budo (the martial arts) in school education in Japan. Arch Budo (2006) 2(1):11–4.

[34] Haugh M. Revisiting the conceptualisation of politeness in English and Japanese. Multilingua-journal Cross-cultural Interlanguage Communication (2004) 23:85–109. doi: 10.1515/mult.2004.009

[35] Yuasa Y Nagatomo S Hull MS. The Body, Self-Cultivation, and Ki-Energy. USA: State University of New York Press (1993).

[36] Blomqvist Mickelsson T. Brazilian jiu-jitsu as social and psychological therapy: a systematic review. J Phys Educ Sport (2021) 21:1544–52. doi: 10.7752/jpes.2021.0319638

[37] Tiggemann M Williamson S. The effect of exercise on body satisfaction and self-esteem as a function of gender and age. Sex Roles (2000) 43(1):119–27. doi: 10.1023/A:1007095830095

[38] Craft BB Carroll HA Lustyk MK. Gender differences in exercise habits and quality of life reports: assessing the moderating effects of reasons for exercise. Int J Lib Arts Soc Sci (2014) 2(5):65–76. 27668243 PMC5033515

[39] Kavoura A Ryba T Kokkonen M. Psychological research on martial artists: A critical view from a cultural praxis framework. Scand Sport Stud Forum (2012) 3:1–23.

[40] Kowalinski E Schneeberger AR Lang UE Huber CG. Safety through locked doors in psychiatry? Leiden, Niederlande: Brill | mentis (2017) p. 147–62.

[41] Schneeberger AR Kowalinski E Fröhlich D Schröder K Felten von S Zinkler M. Aggression and violence in psychiatric hospitals with and without open door policies: A 15-year naturalistic observational study. J Psychiatr Res (2017) 95:189–95. doi: 10.1016/j.jpsychires.2017.08.017 28866330

[42] Vertonghen J Theeboom M. The social-psychological outcomes of martial arts practise among youth: a review. J Sports Sci Med (2010) 9(4):528–37. PMC3761807 24149778

