# Supplementary material for: Implementation of a Budo group therapy for psychiatric in- and outpatients: a feasibility study
Source: Front Psychiatry. 2024 Feb 2;15:1338484. doi: 10.3389/fpsyt.2024.1338484 (PMC10873915; doi:10.3389/fpsyt.2024.1338484)
Supplement: Supplementary file 1 [file DataSheet_1.pdf]

Supplement

Survey Questions

To assess satisfaction and motivation, the following statements were self-reported on a 5-point Likert scale (with 1 = total disagreement and 5 = total agreement):

- “I was satisfied with the Budo group therapy.”
- “I was motivated during the Budo therapy session.”

To assess intervention effects, the following statements were self-reported on a 5-point Likert scale (with 1 = a lot worse and 5 = a lot better):

- “Physically I feel…”
- “Psychologically I feel…”

To assess perspectives on future use, the following statements were self-reported on a 5-point Likert scale (with 1 = total disagreement and 5 = total agreement):

- “The Budo group was beneficial in the therapeutic setting.“
- “I would like to attend the Budo group therapy multiple times per week.”
- “The Budo group would be beneficial even after the discharge from inpatient treatment.”
